# Photodynamic Antimicrobial Action of Asymmetrical Porphyrins Functionalized Silver-Detonation Nanodiamonds Nanoplatforms for the Suppression of *Staphylococcus aureus* Planktonic Cells and Biofilms

**DOI:** 10.3389/fchem.2021.628316

**Published:** 2021-03-11

**Authors:** Yolande I. Openda, Bokolombe P. Ngoy, Tebello Nyokong

**Affiliations:** ^1^ Institute for Nanotechnology Innovation, Department of Chemistry, Rhodes University, Makhanda, South Africa; ^2^ Département de Chimie, Université de Kinshasa, Kinshasa, Democratic Republic of the Congo

**Keywords:** asymmetrical porphyrins, detonation nanodiamonds, silver nanoparticles, staphylococcal biofilms, photodynamic antimicrobial therapy

## Abstract

New asymmetrical porphyrin derivatives containing a *p*-hydroxyphenyl moiety and *p*-acetylphenyl moieties along with their functionalized silver-detonation nanodiamonds nanohybrids were characterized and their photophysicochemical properties were established. The study provides evidence that the metalated porphyrin derivatives were red-shifted in absorption wavelength and possessed high singlet oxygen quantum yield comparative to the unmetalated core, thus making them suitable agents for photodynamic antimicrobial chemotherapy. As a result of conjugation to detonation nanodiamonds and silver nanoparticles, these compounds proved to be more effective as they exhibited stronger antibacterial and anti-biofilm activities on the multi-drug resistant *S. aureus* strain due to synergetic effect, compared to Ps alone. This suggests that the newly prepared nanohybrids could be used as a potential antimicrobial agent in the treatment of biofilms caused by *S. aureus* strain.

## Introduction

Microbial biofilms are the main cause of up to 80% of all human bacterial infections (Hu et al., 2018). These biofilms can be described as a community of microbial cells that are firmly embedded into an extracellular polymeric matrix that attaches to a living or non-living surfaces (Hu et al., 2020). Commonly, due to the altered physiological and metabolic conditions of cells living in a biofilm, these infectious pathogens can tolerate 10–1,000 times higher dosage of antibiotics than their planktonic forms (free floating bacteria cells) (Donlan, 2002; Mirzahosseinipour et al., 2020). This makes it more difficult to eradicate bacterial biofilm cells as they develop more resistance to the available antibiotic treatments via various mechanisms (Wang et al., 2019). In addition, eradicating *S. aureus* is challenging not only because of the emergence of antibiotic resistances (such as Methicillin and Vancomycin) but also due to their high ability to form biofilms (Mamone et al., 2016). This results in increased need for the development and the establishment of novel alternative approaches for the suppression of infectious biofilms without causing development of resistance.

Amongst the proposed technics, photodynamic antimicrobial chemotherapy (PACT), known as a physicochemical treatment (Xu et al., 2016), has shown promising results for the management of relevant localized bacterial infections such as chronic wound infections and dental biofilms (Al-Mutairi et al., 2018). This new antimicrobial approach is considered as a favorable alternative to antibiotic therapy because unlike conventional antibiotics, PACT uses a broad spectrum of actions with efficient inactivation of resistant microorganisms and show low mutagenicity (Tim, 2015). This mode of treatment targets various site in bacterial cell, and presents limited damage to the host tissue, but most importantly, no resistance occurs following several treatments (Liu et al., 2015; Ghorbani et al., 2018).

The photodynamic antimicrobial chemotherapy protocol consists on the use of dyes also known as photosensitisers (PS) and light. The principle is based on the localization of the photosensitizer inside the cell especially in the mitochondria (Mahalingam et al., 2018) or on the bacteria wall and not in the neighboring tissue (Schastak et al., 2010). These chromophores are subsequently exposed to low doses of visible light of specific wavelength in the presence of molecular oxygen (Amos-Tautua et al., 2019), generating harmful species, such as reactive nitrogen species (RNS) and reactive oxygen species (ROS) especially singlet oxygen (^1^O_2_), which are toxic and capable of killing target cells (Baltazar et al., 2015). Characteristics including type, concentration, nature and spectral properties of a PS are crucial in the efficacy of PACT (Afrasiabi et al., 2020). Photosensitizers investigated for PACT include phthalocyanines, and porphyrins (Zhang et al., 2019).

Porphyrins (Ps) are among the most widely used PACT photosensitizers for planktonic cells and biofilms inactivation (Mamone et al., 2016). Porphyrins have received great attention as photosensitisers because of their effective singlet oxygen generation ability (Managa et al., 2019). In order to enhance their application in biomedicine, various nano-carriers have been designed in order to enhance their physicochemical properties and reduce aggregation issue. Examples of some of these nano-carriers are carbon nanomaterials such as detonation nanodiamonds (DNDs) and silver nanoparticles (Ag NPs). DNDs have core-shell structural design with diamond inner core (sp^3^ carbon atoms) and graphitic outer shell (sp^2^ carbon atoms) with hanging bonds ended with functional groups, which include carboxylic acid groups, anhydride, hydroxyl groups and epoxide groups. Due to the presence of carboxylic groups, DNDs suspensions are stable in water and have the capability of complexing with water soluble drugs which is an advantage over other carbon nanomaterials (Chauhan et al., 2020).

Therefore, to the best of our knowledge we report for the first time on the synthesis of new asymmetrical ester containing free base, zinc (II), gallium (III), and indium (III) porphyrins as well as their novel nanoplatforms obtained via covalent linking of the porphyrins to DNDs and colloidal Ag NPs to enhance PACT activities of the Ps by synergetic effect in this work. Asymmetrical porphyrins are employed since asymmetry is known to result in high singlet oxygen quantum yield in porphyrins (Oliveira et al., 2009). In addition, AgNPs and DNDS used in this work are also known for their antimicrobial potency (Szunerits et al., 2016; Grün et al., 2018). A metal free porphyrin have been linked to Ag NPs (Ag-S bond), for antimicrobial studies (Elashnikov et al., 2019). In this work we employ porphyrins containing heavy metals (Zn, In, Ga), which will improve PACT activity. Porphyrins have also been linked to DNDs, but not for PACT (Picollo et al., 2019), and they are applied for PACT for the first time in this work.

## Experimental Section

### Materials

4-Hydroxybenzaldehyde, methyl 4-formylbenzoate, pyrrole, Zinc tetraphenyl-porphyrin (ZnTPP), anhydrous zinc acetate, anhydrous indium (III) chloride, gallium (III) chloride and *N*,*N*’-dicyclohexylcarbodiimide (DCC) and *N*-hydroxysuccinimide (NHS), tryptic soy broth, crystal violet and 9,10 dimethylanthracene (DMA) were purchased from Sigma-Aldrich.

The synthesis of silver nanoparticles was done following a procedure described in literature (Agnihotri et al., 2014). Detonation nanodiamonds (DNDs) were obtained from the Nanocarbon Research Institute Ltd.

Dimethylformamide (DMF), dimethylsulfoxide (DMSO) and all other reagents were obtained from commercial suppliers and were of analytical grade purity and used without further purification. For sample purification, silica gel 60 (0.04–0.063 mm) was used for column chromatography.

The antibacterial experiments were carried out using *Staphylococcus aureus* obtained from Davies Diagnostics, South Africa. The nutrient agar and nutrient broth used to evaluate the bacteria growth and to feed the biofilms were purchased from Merk (Pty) Ltd. South Africa. Phosphate buffer saline (10 mM PBS, pH 7.4) was prepared using appropriate amounts of Na_2_HPO_4_ and NaOH using highly purified H_2_O from ELGA, Veolia water PURELAB, Flex system (Marlow, United Kingdom).

### Equipment

^1^H NMR measurements were performed in a Bruker® AVANCE 600 MHz NMR spectrometer using deuterated DMSO as a solvent. Mass spectra were recorded on Brucker AutoFLEX III Smartbeam TOF/TOF Mass spectrometer using a positive ion mode using alpha-cyano-4-hydroxycinnamic acid as a MALDI matrix. Infrared spectroscopy was carried on using a Bruker Alpha IR (100 FT-IR) spectrophotometer with universal attenuated total reflectance (ATR), whereas a Shimadzu UV-2250 spectrophotometer was used to record all the ground state absorption spectra and the singlet oxygen quencher degradation.

A Varian Eclipse spectro-fluorimeter was employed to measure the fluorescence excitation including emission in solution, and the triplet lifetimes were determined using a laser flash photolysis system consisting of an LP980 spectrometer with a PMT-LP detector and an ICCD camera (Andor DH320T-25F03) and they were determined by exponential fitting of the kinetic curve using Origin Pro 8 software.

To determine the singlet oxygen production of the compounds, photo-irradiations were done using a Spectra-Physics Quanta Ray Indi-40–10 (118 mJ @ 355 nm, 7 ns, 10 Hz) Nd:YAG laser to pump a Spectra-Physics primoScan OPO (405–2,855 nm, 39 mJ @ 430 nm). The irradiation wavelength was determined to be the crossover wavelength between the respective samples and the ZnTPP used as standard.

The size and morphologies of the synthesized compounds were assessed by transmission electron microscopy (TEM) using a Zeiss Libra model 120 operated at 100 kV and an INCA PENTA FET coupled to the VAGA TESCAM using 20 kV accelerating voltage. Thermogravimetric analysis (TGA) was carried out using a Perkin Elmer TGA 800 instrument.

Merck Eppendorf centrifuge 5,810 was used for precipitates extraction, and HERMLE Z233M-2 centrifuge was used for the harvesting of the bacteria cells. PRO VSM-3 Labplus Vortex mixer was used for the homogenization of the bacteria suspension. A thermostatic Oven was used for incubation processes. The Optical density of the bacteria was determined using the LEDETECT 96, a scanner from Interscience for Microbiology Scan® 500 was used to evaluate the colony forming units CFU/ml of the bacteria and for the biofilms quantification. To quantify the biofilms, crystal violet was used as staining agent. The absorbance of the 96-well plates were read at an excitation wavelength of 590 nm using a Synergy 2 multimode microplates reader (BioTek1). Irradiation for PACT studies was conducted using LED shinning at 415 nm.

### Synthesis

**SCHEME 1:**
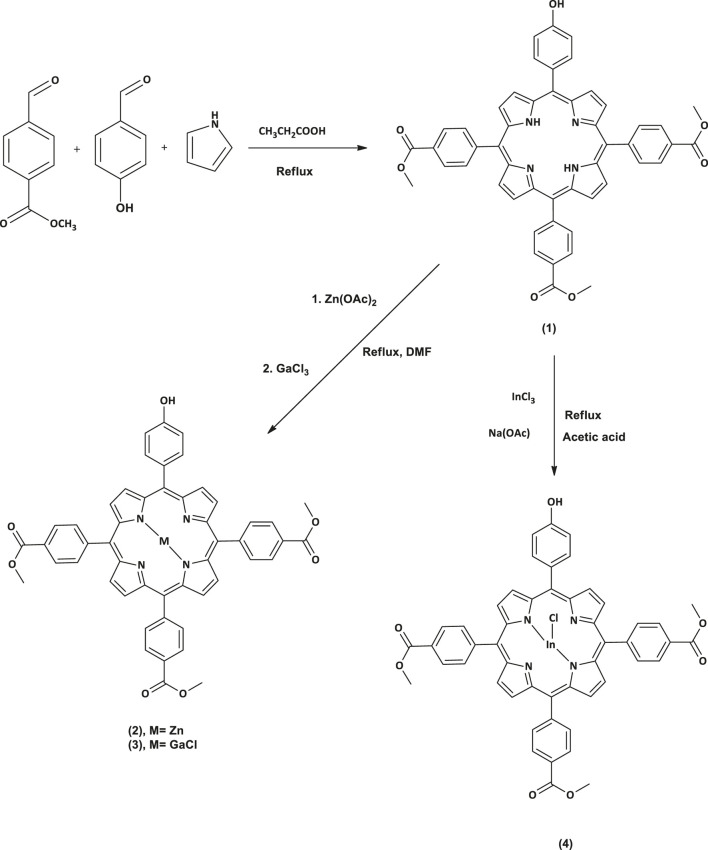
The synthesis of asymmetrical porphyrins **1**, **2**, **3** and **4** in DMSO.

All the reactions were carried out under argon atmosphere. A modified procedure for asymmetrical porphyrins synthesis (Zięba et al., 2012) was applied in this work to prepare complex **1** (Scheme 1). Afterward, compound **1** was used as starting material to synthesize the metalated complexes **2**, **3**, and **4** (Scheme 1) through a metalation reaction which was achieved by inserting different metals, zinc (II), gallium (III) and indium (III), into the porphyrin core. The reaction completion was monitored using UV-Vis spectrometry to check the absence of two of the four Q-bands exhibited previously in the spectrum of complex **1**. The MALDI-TOF MS data and ^1^H NMR data were found to be in full agreement with the proposed structures.

#### 5-(4-Hydroxyphenyl)-tris-10, 15, 20-(4-acetylphenyl)-porphyrin (1)

In a three necked flask equipped with two dropping funnels and a condenser, propionic acid (100 mL) was refluxed at 140°C for 30 min. Then a mixture of 4-hydroxybenzaldehyde (0.5 g, 4.09 mmol), methyl 4-formylbenzoate (2 g, 12.18 mmol) in propionic acid and a pyrrole (1.34 ml, 19.31 mmol) in 6 ml *n*-toluene were simultaneously added dropwise in the three necked flask for a period of 15 min and this was left to stir at refluxing temperature for 2 h. Afterward, the temperature was cooled to 50–60°C and methanol (30 ml) was added and left to stir for another 30 min. The resulting solution was filtered under vacuum and the obtained crude product was washed with methanol and then dried for 30 min in the oven at 60°C. The desired pure compound was obtained as a purple solid after silica gel column chromatography using dichloromethane as an eluent.

Yield: 362 mg (11%). FT-IR: ν, cm^−1^ 3.394 (O-H stretch), 2.999 (Alph. C-H stretch), 2.916 (Ar. C-H stretch), 1.716 (ester C=O stretch), 1.602 (C=N and C=C stretches), 1.432 (C-H bend), 1.270 (ester C-O stretch), 1.104 (C-N stretch), and 959 (=C-H bend). ^1^H NMR (600 MHz, DMSO-*d*_*6*_): δ_H_, ppm 10.01 (*s*, 1H, -OH), 8.93 (*d*, *J* = 8 Hz, 2H, Ar-H), 8.81 (*d*, *J* = 8 Hz, 6H, Ar-H), 8.38 (*d*, *J* = 8 Hz, 6H, Ar-H), 8.34 (*d*, *J* = 8 Hz, 6H, pyrrole-H), 8.00 (*d*, *J* = 8 Hz, 2H, Ar-H), 7.21 (*d*, *J* = 8 Hz, 2H, pyrrole-H), 4.04 (*s*, 9H, -OCH_3_), and 2.54 (*s*, 2H, -NH_2_). MALDI TOF-MS, calc. 804.26, found 804.42 [M]^+^.

#### Zinc 5-(4-Hydroxyphenyl)-tris-10, 15, 20-(4-acetylphenyl)-porphyrin (2)

Complex **1** (0.1 g, 0.12 mmol) was added into a refluxing DMF. The reaction mixture was left to stir while heating until complete dissolution of the starting material. Then, anhydrous zinc acetate (0.078 g, 0.44 mmol) was added and the mixture was continuously heated at 100°C until completion which was monitored using a UV/Vis spectrophotometry. At the completion, the reaction was left to cool at room temperature followed by the addition of ethanol/water mixture (50 ml, 1:1 v/v) to precipitate out the zinc porphyrin (**2**) which was filtered off, washed with Millipore water and dried in vacuo.

Yield: 93.1 mg (90%). FT-IR: ν, cm^−1^ 3.395 (O-H stretch), 3.003 (Alph. C-H stretch), 2.918 (Ar. C-H stretch), 1.714 (ester C=O stretch), 1.656 (C=N and C=C stretches), 1.434–1.409 (C-H bend), 1.314–1.276 (ester C-O stretch), 1.106 (C-N stretch) and 950 (=C-H bend). ^1^H NMR (600 MHz, DMSO-*d*_*6*_): δ_H_, ppm 10.09 (*s*, 1H, -OH), 9.12 (*d*, *J* = 8 Hz, 2H, Ar-H), 9.04 (*d*, *J* = 8 Hz, 4H, pyrrole-H), 8.95 (*d*, *J* = 8 Hz, 2H, Ar-H), 8.45 (*d*, *J* = 8 Hz, 6H, Ar-H), 8.38 (*d*, *J* = 8 Hz, 6H, Ar-H), 8.01 (*d*, *J* = 8 Hz, 2H, pyrrole-H), 7.20 (*d*, *J* = 8 Hz, 2H, pyrrole-H), and 4.01 (*s*, 9H, -OCH_3_). MALDI TOF-MS, calc. 866.17, found 866.61 [M]^+^.

#### Chloro Gallium 5-(4-Hydroxyphenyl)-tris-10, 15, 20-(4-acetylphenyl)-porphyrin (3)

Complex **3** was prepared using a similar procedure as described for complex **2**, except complex **1** (0.1 g, 0.12 mmol) and gallium chloride (0.076 g, 0.44 mmol) were used.

Yield: 105 mg (97%). FT-IR: ν, cm^−1^ 3.374 (O-H stretch), 2.996 (Alph. C-H stretch), 2.776 (Ar. C-H stretch), 1.718 (ester C=O stretch), 1.604 (C=N and C=C stretches), 1.466–1.434 (C-H bend), 1.345–1.274 (ester C-O stretch), 1.107 (C-N stretch) and 952 (=C-H bend). ^1^H NMR (600 MHz, DMSO-*d*_*6*_): δ_H_, ppm 10.11 (*s*, 1H, -OH), 9.14 (*d*, *J* = 8 Hz, 2H, Ar-H), 9.04 (*d*, *J* = 8 Hz, 4H, pyrrole-H), 8.95 (*d*, *J* = 8 Hz, 2H, Ar-H), 8.44 (*d*, *J* = 8 Hz, 6H, Ar-H), 8.38 (*d*, *J* = 8 Hz, 6H, Ar-H), 8.01 (*d*, *J* = 8 Hz, 2H, pyrrole-H), 7.25 (*d*, *J* = 8 Hz, 2H, pyrrole-H), and 4.06 (*s*, 9H, -OCH_3_). MALDI TOF-MS, calc. 906.14, found 906.49 [M]^+^ and 871.48 [M-Cl]^+^.

#### Chloro Indium 5-(4-Hydroxyphenyl)-tris-10, 15, 20-(4-acetylphenyl)-porphyrin (4)

Complex **1** (0.1 g, 0.12 mmol) was dissolved in glacial acetic acid (20 ml) and the mixture stirred and refluxed at 100°C. Indium Chloride (0.138 g, 0.62 mmol) and sodium acetate (0.356 g, 4.4 mmol) were then added to the reaction mixture and left to reflux for 18 h. Afterward, the reaction mixture was cooled to room temperature then poured in cold water to obtain complex 4 as a green-purple precipitate, which was filtered off, severally washed with water and dried in vacuo.

Yield: 77 mg (67%). FT-IR: ν, cm^−1^ 3.384 (O-H stretch), 3.085 (Alph. C-H stretch), 2.951 (Ar. C-H stretch), 1.717 (ester C=O stretch), 1.603 (C=N and C=C stretches), 1.529–1.434 (C-H bend), 1.350–1.274 (ester C-O stretch), 1.105 (C-N stretch) and 966 (=C-H bend). ^1^H NMR (600 MHz, DMSO-*d*_*6*_): δ_H_, ppm 10.11 (*s*, 1H, -OH), 9.14 (*d*, *J* = 8 Hz, 2H, Ar-H), 9.04 (*d*, *J* = 8 Hz, 4H, pyrrole-H), 8.95 (*d*, *J* = 8 Hz, 2H, Ar-H), 8.45 (*d*, *J* = 8 Hz, 6H, Ar-H), 8.39 (*d*, *J* = 8 Hz, 6H, Ar-H), 8.01 (*d*, *J* = 8 Hz, 2H, pyrrole-H), 7.26 (*d*, *J* = 8 Hz, 2H, pyrrole-H), and 4.07 (*s*, 9H, -OCH_3_). MALDI TOF-MS, calc. 952.12, found 953.41 [M+H]^+^ and 918.38 [M-Cl+H] ^+^.

#### Nanoconjugation of Porphyrin Complexes to DNDs and Ag NPs

**SCHEME 2:**
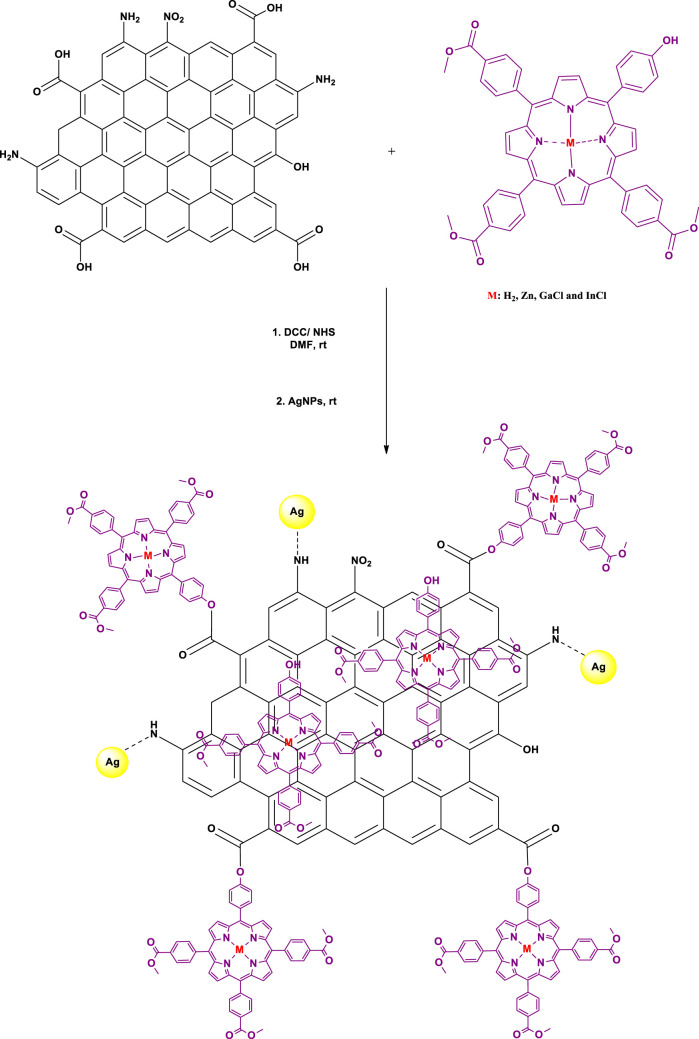
The synthesis of Ps-DNDs@Ag nanohybrids from DNDs, Ps and AgNPs.

Firstly, an ester covalent bond between Ps and DNDs was formed according to a previously reported method (Matshitse et al., 2019), taking advantage of the OH group on the Ps and the COOH from DNDs to result in Ps-DNDs nanohybrids (Scheme 2). Briefly, DNDs (10 mg) were dissolved in 3 ml dry DMF and DCC (0.010 g, 0.049 mmol) was added separately to activate COOH groups on DNDs with continuous stirring for 48 h. Thereafter, respective Ps (10 mg each) and NHS (0.008 g, 0.07 mmol) as well as the bare AgNPs (5 mg) were subsequently added to the respective vessels and were allowed to react while stirring at room temperature for another 72 h. It is important to note that physical interactions will take place between AgNPs and nitrogen atoms on DNDs through Ag-nitrogen affinity.

The mixtures were repeatedly centrifuged at 3,500 rpm for 5 min in ethanol to remove the unreacted starting materials and the obtained precipitates were air dried in the fume hood. The formed nanoconjugates are represented as **1**-DNDs@Ag, **2**-DNDs@Ag, **3**-DNDs@Ag and **4**-DNDs@Ag.

### PACT Studies

#### Antibacterial Assays on Planktonic Cells

The antibacterial effect of the newly prepared Ps and the nanoconjugates was tested on the Gram positive bacteria S*taphylococcus aureus* ATCC 25923. The later was prepared according to protocols described in literature with slight modifications (Openda et al., 2020a). Briefly, an aliquot of *S. aureus* was aseptically added into 5 mL of newly prepared agar broth and the suspension was allowed to incubate at 37°C while shaking until a mid-logarithmic phase (OD 0.6–0.8 at 620 nm) was observed. Thereafter, the bacterial suspension was centrifuged (4000 RPM for 10 min) and washed three times with PBS. The resulting *S. aureus* pallet was resuspended in 100 ml of PBS to make a dilution factor of 10^–2^ bacterial suspension stock. Afterwards, serial dilutions of 10^–3^, 10^–4^, 10^–5^, 10^–6^, and 10^–7^ were prepared for the bacteria optimization. For this, 100 µL from each dilution were aseptically inoculated on agar plates in triplicates, followed by 18 h incubation at 37°C. Then a CFU counting was performed and the dilution factor of 10^–6^, corresponding to a count with 3.02 × 10^10^ CFU/mL), was practically the best to be used for this study. The concentration of 10^–6^ was chosen as working concentration since the plates contained an average of 377 colonies.

PACT was carried on following a slightly modified procedure (Makola et al., 2020). For this, 1% DMSO/PBS was used to dissolve all the samples. 2.5, 5, 10, 20, and 40 μg/mL were prepared to optimize the Ps concentration and the optimal concentration for Ps was found to be 10 μg/ml. Light emitting diode M415L4 (LED) was used as a light source at a constant irradiance of 15.6 μW/mm^2^ at 415 nm excitation wavelength for 2 h with 30 min intervals.

#### Biofilm Formation and Photodynamic Assays

The single-species biofilm of *S. aureus* was formed with slight modifications (Openda et al., 2020b) as follows: Newly prepared inocula of the bacterial species at 10^8^ CFU/mL was diluted to 10^9^ CFU/mL using tryptic soy broth. 200 μl of the suspension were seeded in 96 well plates and put to incubate statically and anaerobically at 37°C for 5 days, while regularly removing unbound cells after every 18 h by gently washing with PBS and refilling the wells with 200 μL fresh tryptic soy broth to stimulate biofilm formation.

At the end of the incubation period, the biofilm-coated wells were carefully washed twice with PBS and left to air dry for 30 min.

For PACT assays, 100 μL of each sample were added into the 96-well plates containing biofilms with different concentrations of 25, 50, and 100 μg/mL). The suspensions were after that allowed to incubate for 30 min in the dark at 37°C, before irradiating at 415 nm excitation wavelength for 30 min with a LED. After irradiation, on one hand, to quantify the biofilms survival, 200 μl of 1% aqueous crystal violet (CV) solution was added into the 96-well plate to stain the cells for 30 min. Afterwards, the excess CV was washed off thrice with PBS and the wells were refilled with PBS for the cell survival counting.

On the other hand, the suspensions were diluted 1,000 times in PBS, and 100 μL of each sample were aseptically inoculated on agar plates then incubated for 18 h at 37°C to determine the number of CFU/mL. The same was also done for the samples kept in dark and all tests were performed in triplicate and the wells with bacterial films in 1% DMSO/PBS served as a positive control.

### Statistical Analysis

Three independent (*n* = 3) experiments were done in triplicates. Triplicate measurements were made to ensure accuracy and the results were compared by using a 3-way factorial ANOVA. The data were presented as means ± standard deviation (SD) of log_10_ CFU values for the planktonic cells or means ± standard deviation (SD) of cell survival for the biofilms. A *p*-value of 0.05 was considered statistically significant.

## Results and Discussion

### Synthesis and Characterization

In this study, we synthesized the unsymmetrical derivatives Ps **2**, **3** and **4** by introducing Zn, GaCl and InCl into the core of the free base Ps **1** (Scheme 1), respectively. Afterwards nanohybrids were acquired by linking the Ps to the DNDs via ester bonds, then nitrogen atoms on the DNDs and Ag NPs were linked via physical interactions (Scheme 2), to get more enhanced photoantimicrobial results due the synergetic effects. The compounds were characterized by NMR, IR, UV-Vis, MALDI-TOF MS, TEM, and TGA (see Supplementary Figures S1–S6 in the SI for MS and NMR) and the acquired data were consistent with the predicted structures.

### UV-Vis Spectroscopy

**FIGURE 1:**
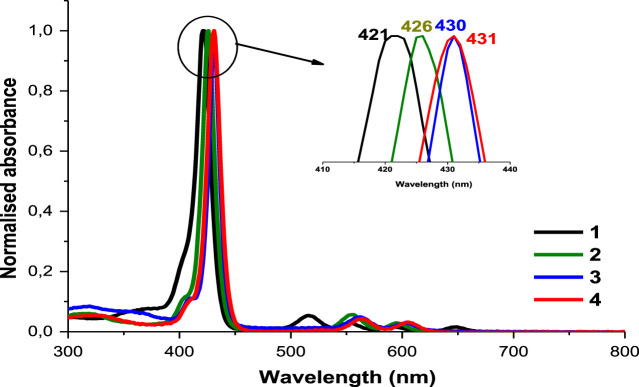
The normalized absorption spectra of asymmetrical porphyrins **1**, **2**, **3** and **4** in DMSO. Arrow shows expansion of the B bands.

As explained by Gouterman’s four orbital model, porphyrins optical spectra usually contain a very intense Soret or B-band rising around 400 nm and multiple Q bands observed between 500 and 600 nm (Gouterman 1978; Huang et al., 2015). Figure 1 shows typical absorbance spectra of porphyrins. The B-band of **1** appears at 421 nm (Table 1) with four Q-bands in DMSO. Following the insertion of respective metals in compound **1**, the B-bands of complexes **2**, **3** and **4** appeared red-shifted at 426, 430, and 431 nm, respectively (Table 1), as the four Q-bands collapsed into two. The observed spectral red-shifts of the Soret bands results from the heavy metal effect which could cause a degree of perturbation and electron delocalisation within the porphyrin macrocycle (Uttamlal and Holmes-Smith, 2008). Indium derivatives showed the largest shifts due to the non-planar effect of the In (III) ion and its bigger atomic radius compared to Zn (II) and Ga (III) ions (Gürel et al., 2015).

**TABLE 1:** Photophysicochemical data of synthesized complexes in DMSO.

Complex^a^	λ_abs_ (nm)	λ_em_ (nm)	Φ_F_	τ_T_ (μs)	Φ_Δ_
**1**	426	657,719	0.074	283	0.27
**2**	430	615,664	0.069	232	0.43
**3**	431	610,662	0.033	201	0.48
**4**	422	607,652	0.021	182	0.54
**1**-DNDs@Ag (13)	429	657,721	0.048	185	0.33
**2**-DNDs@Ag (20)	431	610,655	0.036	180	0.51
**3**-DNDs@Ag (24)	431	606,658	0.015	176	0.58
**4**-DNDs@Ag (31)	426	605,655	0.021	140	0.59

^a^ TEM sizes (nm) in brackets. DNDs = 2.4 nm, Ag NPs = 7.0 nm.

**FIGURE 2:**
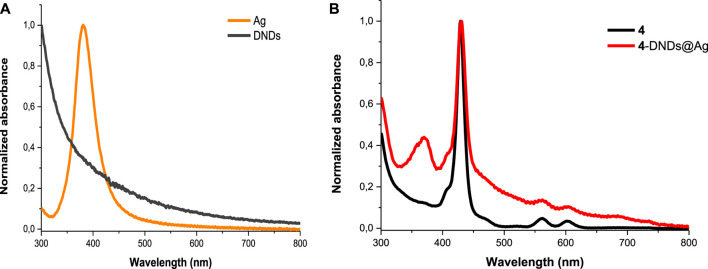
Electronic absorption spectra of **(A)** DNDs and Ag NPs and **(B) 4** and **4**-DNDs@Ag in DMSO (as an example).

In Figure 2A, DNDs showed a broad feature with no absorption peak in the visible region, however the Ag NPs showed a surface plasmon resonance (SPR) band at 380 nm. In the conjugates spectra, the increased absorption in the region below 410 nm indicates the absorbance of the SPR band of Ag NPs which has absorption wavelength almost close to the Soret band of the porphyrins but also the presence of DNDs (Figure 2B and Supplementary Figures S7,S8). The slight spectral red-shifts in the Soret and Q-bands maxima for Ps in 1-DNDs@Ag (422 nm), 2-DNDs@Ag (429 nm), 3-DNDs@Ag (431 nm), and 4-DNDs@ Ag (431 nm) following conjugation (Scheme 1) are brought by J aggregation. These slight red shifts were also seen in the tetrasulfonated zinc Pc-graphene complex and were related to a J type aggregation (Zhang and Xi, 2011). However, in the nanoconjugates UV-Vis spectra, the enhancement of absorbance observed especially in the region below 450 nm indicate the presence of the nanoparticles, Supplementary Figures S7,S8 used as examples.

The mass loading of the Ps onto the nanohybrids was calculated using UV-Vis absorption technic This method consist of comparing the Q band absorbance intensities of the nanohybrids to that of the respective Ps alone. Thus, equal masses (mg) for Ps alone and their respective conjugates were separately weighed and dissolved in the same volume of solvent. The respective masses were 893, 854, 802, and 747 μg (Ps)/mg DNDs@Ag for **1**-DNDs@Ag, **2**-DNDs@Ag, **3**-DNDs@Ag and **4**-DNDs@Ag, respectively. The observed higher loadings are most likely due to the presence of both π-π interactions and the ester bond. **1**-DNDs@Ag showed the highest loading due to the absence of central metal whose size can limit strong interactions between the molecule and the DNDs surface.

### FT-IR Spectra Analysis

**FIGURE 3:**
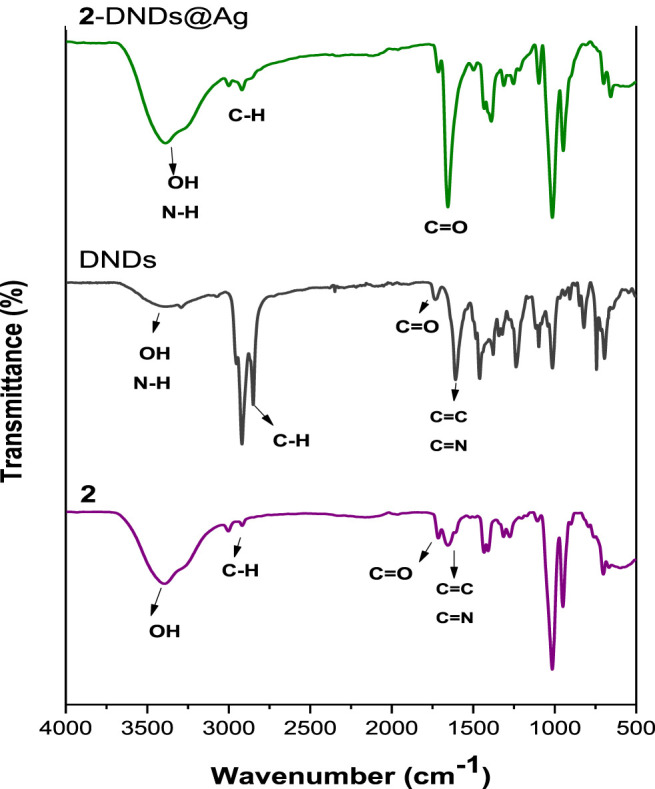
The FT-IR spectra of **2**, DNDs, **2**-DNDs@Ag (used as an example).

The formation of the ester linkage between the porphyrins and the DNDs was confirmed by FT-IR spectra (Figure 3 and Supplementary Figure S9). The spectral analysis of Ps alone indicates the presence of characteristic vibration peaks at about 3,395–3,374 cm^−1^ and 1716 cm^−1^ for O-H and ester-C=O stretches, respectively. These characteristic peaks of the Ps alone cannot be distinguished because they overlap with the characteristic peaks of the nanoconjugates.

Hence the successful crafting of Ps to the DNDS can be confirmed by the evident increase in the intensity of OH and C=O groups as well as their shifts to higher frequencies in the spectra of the conjugates, suggesting a high predominance of these groups in the as prepared nanohybrids since they contain both the Ps and the DNDs that also possess -OH and carboxylic C=O groups. Shifts in FT-IR bands confirm molecular interactions.

### Transmission Electron Microscopy (TEM)

**FIGURE 4:**
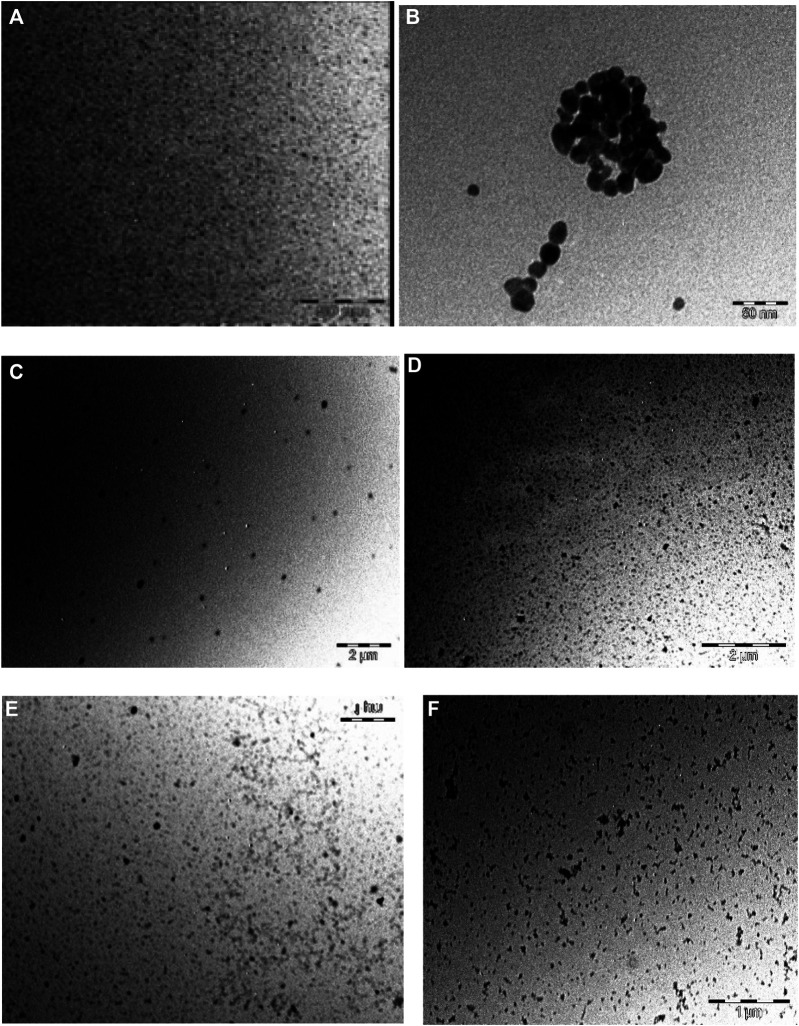
TEM images of **(A)** DNDs, **(B)** Ag NPs, **(C) 1**-DNDs@Ag, **(D) 2**-DNDs@Ag, **(E) 3**-DNDs@Ag and **(F) 4**-DNDs@Ag (showing the size and morphology).

As noticed, the acquired images in Figure 4 show that the DNDs and the silver NPs were spherical with an average size of 2.4 nm and 7 nm, respectively. The size increased as a result of aggregation caused by conjugation to complexes. The size became ∼ 13, 20, 24, and 31 nm for **1**-DNDs@Ag, **2**-DNDs@Ag, **3**-DNDs@Ag and **4**-DNDs@Ag, respectively, Table 1.

### Thermogravimetric Analysis (TGA)

TGA analysis was carried out to ascertain the thermal stability of DNDs before and after linkage to Ps and capping with silver nanoparticles. To determine the thermal stability of these compounds, the weight loss was estimated as a function of temperature. To do so, the experiments were recorded at atmospheric pressure under nitrogen flow at a temperature from 50 up to 1000°C with a heating rate of 10°C/min.

**FIGURE 5:**
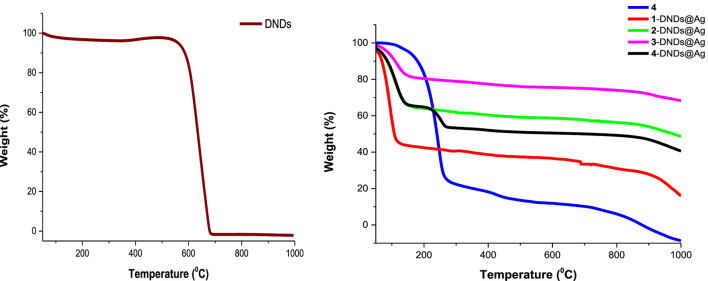
TGA thermograms of DNDs, complex **4** and the nanoconjugates showing thermal stability after conjugation.

The results prove that DNDs started to decompose around 560°C showing a weight loss of 95.03%. This agrees with literature data confirming that nanodiamonds graphitization begins nearby 600°C (Efremov et al., 2018). In comparison to the nanohybrids, at 560°C, the weight loss was of 35%, 58%, 75%, and 70% for **1**-DNDs@Ag, **2**-DNDs@Ag, **3**-DNDs@Ag, and **4**-DNDs@Ag, respectively. Figure 5 (used as an example) illustrates that upon functionalizing the DNDs with complex **4** (as an example) and the Ag NPs, decreased weight loss was observed compared to the DNDs alone 95%, thus indicating improvement in thermal stability of DNDs. Similar trends were also observed for DNDs functionalised with silicon phthalocyanines (Matshitse and Nyokong, 2020) and for single walled carbon nanotubes (SWCNTs) following their functionalization to zinc monocarboxyphenoxy phthalocyanine spermine (Ogbodu et al., 2015).

Complete weight loss (100%) of the DNDs was observed around 679°C and at this temperature the conjugates lost weight was 37%, 59%, 76%, and 51% for **1**-DNDs@Ag, **2**-DNDs@Ag, **3**-DNDs@Ag, and **4**-DNDs@Ag, respectively as shown in Figure 5.

### Photophysicochemical Properties

Photophysicochemical parameters such as fluorescence quantum yield (Φ_**F**_), triplet lifetime (τ_T_) and singlet oxygen quantum yield (Φ_**Δ**_) were investigated using comparative methods as described in literature with equations shown in the supporting information. ZnTPP was used as a standard in DMSO with Φ_**F**_ = 0.030 and Φ_**Δ**_ = 0.53 (Makola et al., 2020) as obtained in DMF.

#### Emission Spectra and Fluorescence Quantum Yield (Φ_F_)

**FIGURE 6:**
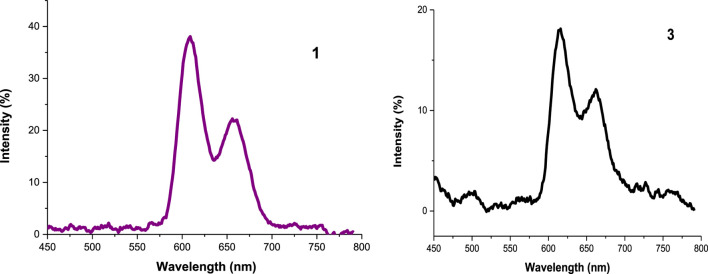
The emission spectra of **1** and **3** in DMSO.

All the experiments were run in DMSO and the absorbance of the photosensitizers at the excitation wavelength was 0.05. The emission spectra showed two characteristic bands of porphyrins (Uttamlal and Holmes-Smith, 2008; Topkaya et al., 2017 **)** as seen in Figure 6 (as an example), Table 1 and in Supplementary Figure S10. The decrease in fluorescence intensities for the metalated Ps and the nanoconjugates could be supported by the heavy central metal effects (Nyokong, 2007) and the presence of electron donating groups on the DNDs favor intersystem crossing process to the triplet state over fluorescence process (De Souza et al., 2016). Additionally, the high aggregation observed in the nanoconjugates contribute to transform electronic excitation energy to vibrational energy, thus resulting in less fluorescent compounds (Muthukumar et al., 2016). Also, Ag NPs have been reported to quench fluorescence (Rapulenyane et al., 2013). Hence, lower Φ_**F**_ values listed in Table 1 were obtained for metalated Ps-DNDs@Ag for the reason above mentioned. A similar trend has previously been observed for phthalocyanines-like complexes (Openda et al., 2020b).

### Transient Spectroscopy

**FIGURE 7:**
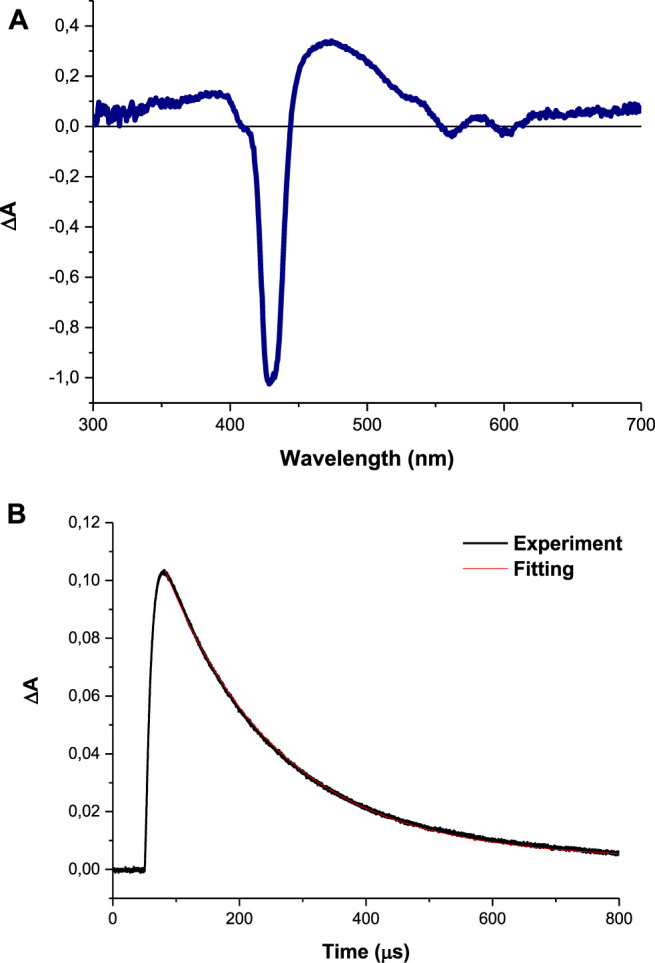
**(A)** Absorption spectra obtained with LFP at 1 μs after excitation and **(B)** transient decay curve (using complex 4 as an example) observed at 425 nm in DMSO.

Nanosecond laser flash photolysis of compound **4** gave a transient absorption spectrum with a strong maximum around 432 nm shown in Figure 7A with a decay lifetime curve in degassed DMSO solution (Figure 7B). The transient absorption spectra and the decay lifetime were attributed to the triplet state of the studied compounds. Triplet state absorption spectra and lifetimes for some selected porphyrins were reported to be in the range of 430–470 nm and microsecond respectively (Barbosa Neto et al., 2013).

The triplet lifetime (τ_T_) can be defined as the time that the excited molecules spend in the triplet state before returning to the ground state and the energy transfer efficiency to molecular oxygen. In the present study, the samples were dissolved in DMSO then saturated with argon gas. The synthesis of the Ps-DNDs@Ag resulted in decreased τ_T_ values, as shown in Table 1, compared to Ps alone.

The triplet lifetime values (an example of the triplet decay curve is shown in Figure 7) were in the range of 140–283°μs (Table 1). As expected, the triplet lifetimes of the Ps decreased after conjugation to the nanoparticles.

#### Singlet Oxygen Quantum Yield (Φ_Δ_)

Singlet oxygen quantum yields is the main parameter used to evaluate the ability of a photosensitizer to destroy bacteria cells. This reactive oxygen species is formed via an energy transfer process between the excited triplet state of the photosensitizer and the ground state molecular oxygen *via* the so called type II reaction (Çakır et al., 2016).

**FIGURE 8:**
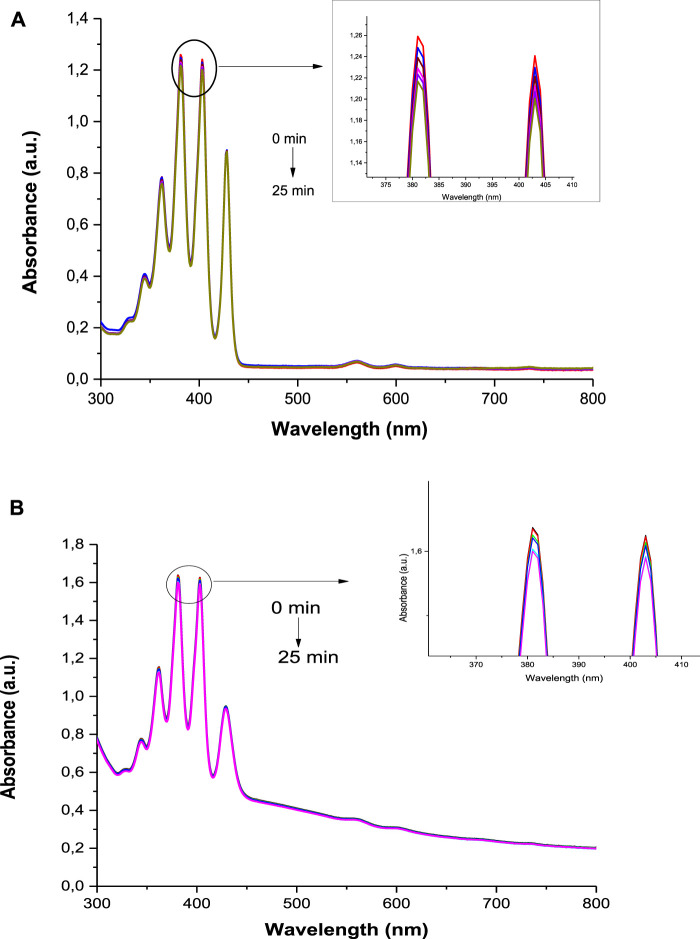
Photobleaching of DMA in DMSO in the presence of **(A)** **3** and **(B)** **3**-DNDs@Ag as examples.

The Φ_Δ_ values were determined by monitoring the photobleaching of DMA in DMSO with irradiation at 424 nm, cross-over wavelength with the ZnTPP standard. DMSO solutions of photosensitizer (1.5 x 10^–5^ M) and DMA (2 x 10^–5^ M) were prepared in the dark under ambient conditions. The irradiation was done at the crossover wavelength with ZnTPP using a laser. Figure 8 (**3**-DNDs@Ag used as an example) and Supplementary Figure S11 confirm the stability of the Ps as no change in the absorption of B bands of the porphyrins was identified. The Φ_Δ_ data are supplied in Table 1 and were determined to be 0.27, 0.43, 0.48 and 0.54 for 1, 2, 3, and 4, respectively, whereas were obtained for 0.33, 0.51, 0.58, and 0.59 for **1**-DNDs@Ag, **2**-DNDs@Ag, **3**-DNDs@Ag, and **4**-DNDs@Ag, respectively.

The higher Φ_Δ_ values were obtained for complexes containing diamagnetic metals, known to have high singlet oxygen quantum yields and long triplet lifetimes which are important for the photoinactivation process (Oluwole et al., 2016). The Φ_Δ_ values were slightly increased for the nanoconjugates when compared to the complexes alone, due to the effect of the nanoparticles, with the reason mentioned above about the presence of electron donating groups on the DNDs as well as the Ag NPs that stimulate the intersystem crossing process to the excited triplet state from where the singlet oxygen is generated. In addition, the presence of a reactive carbonyl group in a molecule is reported to augment singlet oxygen quantum yields because of the n-π^x^ transition are favored by oxygen atom (Wang et al., 2016). The capacity of studied compounds to generate such high singlet oxygen quantum yield makes them good candidates for PACT studies.

### Photodynamic Antimicrobial Studies

For a material to be considered as suitable for PACT applications, it should be able to produce singlet oxygen, and that is the case for the newly synthesized materials in the present work. The bacteria strain of interest is *S. aureus* since it is notorious for high resistance to the commonly used antimicrobial treatments (Chambers and DeLeo, 2009). For the efficient PACT activity on planktonic cells and biofilms of *S. aureus*, in the current work, we developed functionalized DNDs with newly synthesized Ps and Ag NPs that could be able to efficiently bind to the cell-wall, thus to facilitate the penetration of the drug and ability to act within the cell since the generation of singlet oxygen is done within the cell (Tokubo et al., 2018).

#### *In vitro* Antibacterial Activity on Planktonic Cells

**FIGURE 9:**
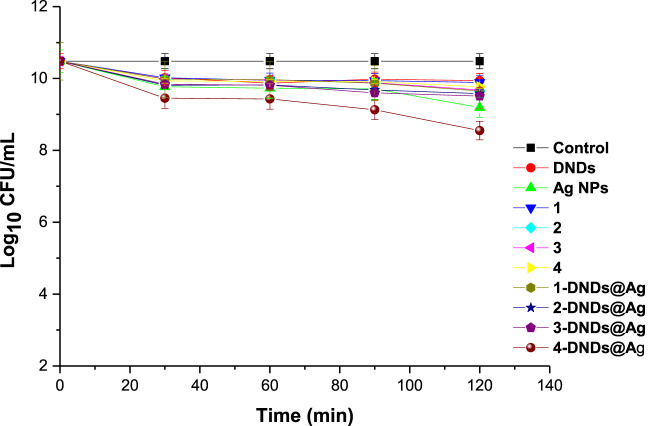
Dark studies of the synthesized compounds on *S. aureus* planktonic cells (irradiation at 415 nm). Concentration = 10 μg/mL. Data represent the mean ± SD (standard deviation).

**FIGURE 10:**
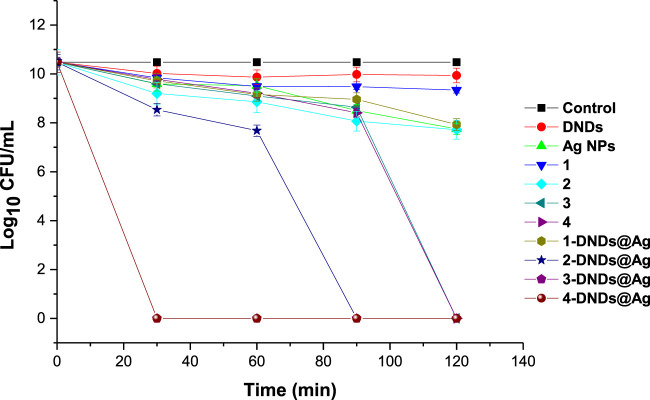
Photoinhibition studies of the synthesized compounds on *S. aureus* planktonic cells (irradiation at 415 nm). Concentration = 10 μg/mL. Data represent the mean ± SD (standard deviation).

The control solutions were prepared with 1% DMSO/PBS without the photosensitizers. And these controls showed no antibacterial effect neither after dark or light treatments as seen in Figures 9,10.

A concentration of 10 μg/mL in 1% DMSO/PBS was prepared for all the samples for comparison reason. Afterwards, the photosensitizers were subjected to dark toxicity and irradiation processes. In this case, no dark toxicity was observed for the samples as Figure 9 exhibited no significant change in log_10_ (CFU, colony forming units) values except for **4**-DNDs@Ag which had a value of 1.93 log_10_ reduction.

For the irradiation processes, as displayed in Figure 10 and data in Table 2, the conjugates of gallium and indium effectively killed the bacteria strains in a short period of time of 30 min with a log reduction of 10.48 ± 0.003 as their complexes counterparts showed full activity at 120 min. These results were expected most likely due to reactive oxygen species such as singlet oxygen and radicals produced by the Ps-DNSs@Ag conjugates. The trend was also confirmed by the high values of singlet oxygen quantum yield which is almost the same for **3**-DNDs@Ag and **4**-DNDs@Ag, Table 1.

**TABLE 2:** Log reduction values for 10 μg/mL of samples in 1% DMSO/PBS after irradiation on *S. aureus*.

Complex	Log reduction	Time of irradiation (min)
**DNDs**	0.54 ± 0.002	120
**Ag NPs**	2.73 ± 0.003	120
**1**	1.13 ± 0.003	120
**2**	2.75 ± 0.005	120
**3**	10.48 ± 0.004	120
**4**	10.48 ± 0.003	120
1-DNDs@Ag	2.54 ± 0.003	120
2-DNDs@Ag	10.48 ± 0.003	90
3-DNDs@Ag	10.48 ± 0.003	30
4-DNDs@Ag	10.48 ± 0.003	30

The zinc conjugate completely inhibited the bacteria at 90 min of irradiation giving a log reduction of 10.48 ± 0.003 yet the zinc porphyrin alone killed gave a log reduction of 2.75 ± 0.005 after 120 min irradiation. For the core, inhibition with log reduction of 1.13 ± 0.003 and 2.54 ± 0.003 were obtained after 120 min for 1 and **1**-DNDs@Ag, respectively. The efficient killing mostly observed for the metalated compounds is expected as the compounds present stronger affinity to the cell-wall thus resulting in complete cell membrane destruction and enhanced drug-cell uptake for efficient photo-antibacterial abilities since they are producing the singlet oxygen in a close proximity of the cell. Also the synergetic effect from the antibacterial activities of Ag NPs which has shown good activity of 2.73 ± 0.003 log_10_ reduction, has contributed to the results.

The log reductions for complexes alone were lower compared to corresponding conjugate derivatives even at a high irradiation time of 120 min, thus showing the importance of the conjugation to nanoparticles, Table 2.

Since PACT process relies on singlet oxygen production by the photosensitizer, the data presented in this work are in perfect agreement with singlet oxygen generated by the compounds. These values also confirm the importance of conjugation of photosensitizers to carbon nanomaterials and AgNPs that have also been reported to possess intrinsic antibacterial properties. The synthesized nanoconjugates showed better PACT activities corresponding to high singlet oxygen quantum yields.

#### *In vitro* Biofilms Eradication

To determine the therapeutic efficacy of the new photosensitizers we carried on studies using three different concentrations of 25, 50, and 100 μg/mL of each of the synthesized compounds for the photo-antibiofilm activity after 30 min of irradiation at 415 nm.

The treatment doses were increased for biofilms compare to planktonic cells studies owing to the lower sensitivity of biofilms toward antimicrobial treatment compared to their planktonic cells counterparts. This is can be explained by the composition of the extracellular polymeric matrixes of biofilms that stops the penetration of drugs within the biofilms (Donnelly et al., 2007; Deng et al., 2019).

**FIGURE 11:**
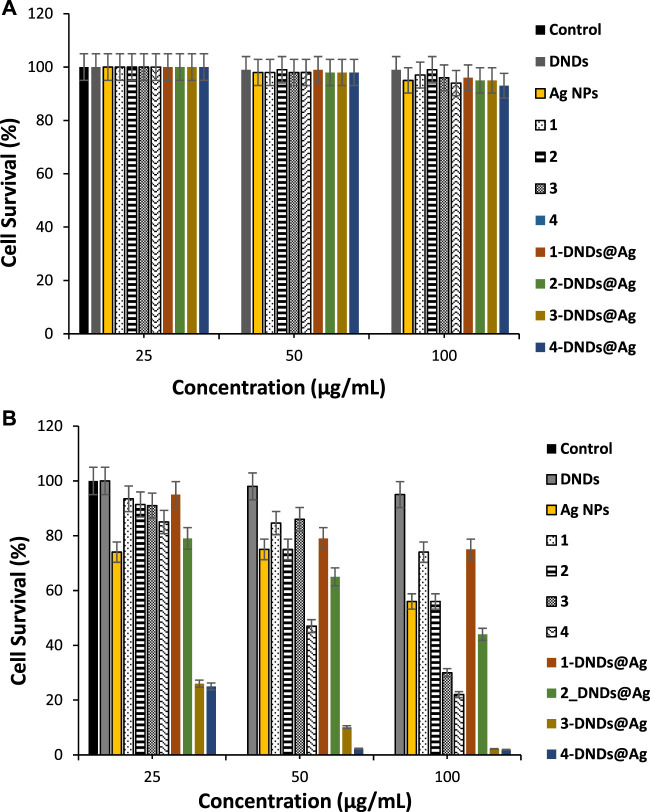
**(A)** Dark toxicity studies and, **(B)** Photoinhibition studies of the synthesized compounds on *S. aureus* biofilms cells (30 min irradiation at 415 nm). Data represent the mean ± SD (standard deviation).

The quantification of biofilms formation by crystal violet assays showed that *S. aureus* is a strong biofilm producer. Figure 11A illustrates that all the compounds did not show dark toxicity activities on the bacteria at all concentrations.

As listed in Table 3, for *S. aureus* biofilms, **3-**DNDs@Ag and ****4**-**DNDs@Ag showed lowest values of 2.20% and 1.92%, photoinhibition of biofilm cells at 100 μg/mL, respectively. While at 50 μg/mL they gave 10.15% and 2.30%, respectively.

**TABLE 3:** Cell survival values of samples in 1% DMSO/PBS after 30 min irradiation on *S. aureus* biofilms.

	Cell survival (%)		
**Complex**	25 μg/mL	50 μg/mL	100 μg/mL
**DNDs**	100.00	99.00	98.00
**Ag NPs**	100.00	98.00	95.00
**1**	93.46	74.50	74.00
**2**	91.40	75.56	56.78
**3**	91.98	86.90	30.84
**4**	95.00	47.20	22.00
1-DNDs@Ag	95.00	79.09	75.60
2-DNDs@Ag	79.00	75.54	44.00
3-DNDs@Ag	26.00	10.15	2.20
4-DNDs@Ag	25.23	2.30	1.92

As displayed in Figure 11B, only the indium and gallium derivatives were able to statistically give significant reductions in biofilm at 100 μg/ml upon 30 min of irradiation. The complexes alone exhibited no major reductions in biofilms in comparison to the respective untreated control groups. The great decrease in cell viability and the successful eradication of the *S. aureus* biofilm species under 30 min light exposition observed specially for the gallium and indium nanoconjugates could be due to the synergistic effect brought by the DNDs, Ag NPs and the Ps alone.

To the best of our knowledge, this work reports for the first time on the photo-eradication of *S. aureus* biofilms using as photosensitizers agents the nanoplatforms containing asymmetrical porphyrins, detonation nanodiamonds and silver nanoparticles. The basic mode of action of the new compounds studied might include inhibition of cell metabolism and growth, damage to the cytoplasmic membrane and increase in cell permeability due the charges found on the moieties (Ribeiro et al., 2013; Rosseti et al., 2014 **)**. All of the obtained data further confirm that the newly prepared nanohybrides could be used as potential photoantibacterial agents against *S. aureus* planktonic cells and biofilms at low concentrations of the complexes with small light doses.

## Conclusion

Novel asymmetrical ester substituted porphyrins and their DNDs and Ag NPs functionalized derivatives are reported for the first time in this work. The prepared compounds proved to be able to generate singlet oxygen and have high potential in the eradication of not only the bacterial planktonic cells of S. aureus, but they also possessed good activities against their difficultly treated bacterial biofilms. In the current study, we also reports on the characterization and photophysicochemical parameters of all the as-prepared compounds. The indium and gallium derivatives were found to have better properties as well as the Ps-DNDs@Ag. In most cases, high singlet oxygen quantum yields were obtained. The photo-antimicrobial activities the complexes/conjugates using PACT with irradiation at 415 nm were also determined on *S. aureus* planktonic and biofilms cells. The obtained data in PACT were all in agreement with the reported photophysicochemical results. The *in vitro* results indicate that at lower concentration the synthesized photosensitisers have a great prospective in biofilms ablation.

## Data Availability

The original contributions presented in the study are included in the article/Supplementary Material, further inquiries can be directed to the corresponding author.
