# Supplementary material for: Photodynamic Antimicrobial Action of Asymmetrical Porphyrins Functionalized Silver-Detonation Nanodiamonds Nanoplatforms for the Suppression of Staphylococcus aureus Planktonic Cells and Biofilms
Source: Front Chem. 2021 Mar 11;9:628316. doi: 10.3389/fchem.2021.628316 (PMC7991625; doi:10.3389/fchem.2021.628316)
Supplement: Supplementary file 1 [file datasheet1.zip › Frontier Figurers/7. (Figure 5).docx]

**Figure 5**
