# Supplementary figures and images for: Photodynamic Antimicrobial Action of Asymmetrical Porphyrins Functionalized Silver-Detonation Nanodiamonds Nanoplatforms for the Suppression of Staphylococcus aureus Planktonic Cells and Biofilms
Source: Front Chem. 2021 Mar 11;9:628316. doi: 10.3389/fchem.2021.628316 (PMC7991625; doi:10.3389/fchem.2021.628316)

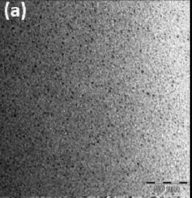

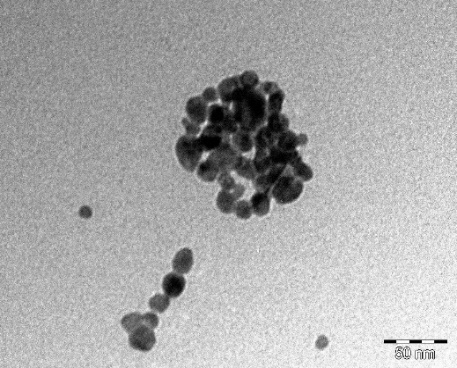


**(c)**

**(e)**

**(b)**


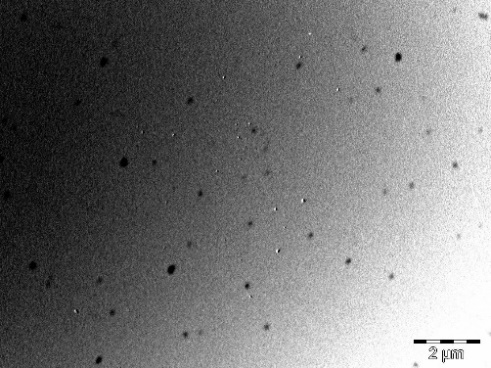
 **
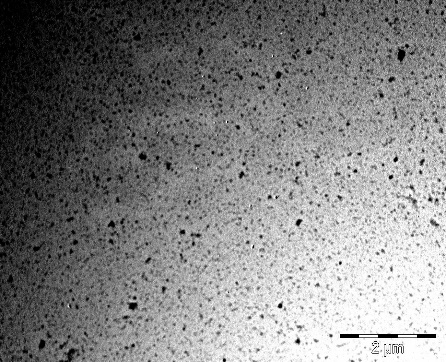
**

**(f)**

**(d)**

**
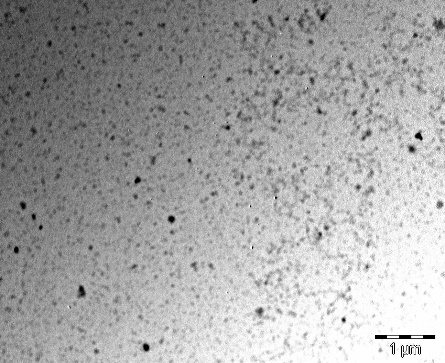

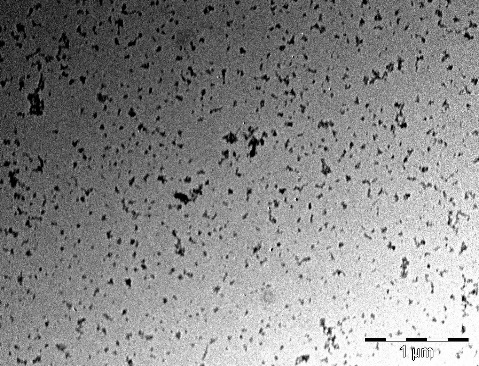
**

**Figure 4**

Supplement: Supplementary file 1 [file datasheet1.zip › Frontier Figurers/6. (Figure 4).docx]
