# Supplementary material for: Photodynamic Antimicrobial Action of Asymmetrical Porphyrins Functionalized Silver-Detonation Nanodiamonds Nanoplatforms for the Suppression of Staphylococcus aureus Planktonic Cells and Biofilms
Source: Front Chem. 2021 Mar 11;9:628316. doi: 10.3389/fchem.2021.628316 (PMC7991625; doi:10.3389/fchem.2021.628316)
Supplement: Supplementary file 2 [file table1.docx]

Supplementary Material

1. **Equations employed**

The fluorescence quantum yield values of the porphyrin complexes alone or their conjugates were recorded using a comparative method reported in literature with equation 1 below (Ogunsipe et al., 2004; Nyokong et al., 2010).


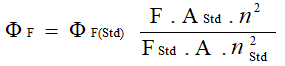
 (1)

In this equation *F* and *F*_Std_ are the area under the emission curves of the sample and standard, respectively. *A* and *A*_Std_ are the absorbance values corresponding to the excitation wavelength of the studied samples and standard, respectively. *n* and *n*_Std_ express the refractive index of the solvents used to prepare the solution of the studied samples and standard, respectively. The ZnTPP was used as a standard with the value: Φ_F_ = 0.030 (Kee et al., 2008).

The determination of the singlet oxygen quantum yields (Φ_Δ_) values of the prepared Ps and nanoconjugates were carried out following a UV/Vis spectroscopic method in DMSO. The monitoring was done spectroscopically following the photobleaching of 9, 10-dimethylanthracene (DMA) as a singlet oxygen scavenger. ZnTPP (Φ_Δ_ = 0.53 in DMSO) **(**Kee et al., 2008) was utilized as standard for comparative purpose using equation 2.

Φ_Δ_ = ${}_{\Delta}^{Std}\frac{R I_{abs}^{Std}}{{R^{Std}I}_{abs}}$ (2)

Where ${}_{\Delta}^{Std}$is the singlet oxygen quantum yield for the standard, R and R^Std^ are the DMA photobleaching rates in the presence of Ps derivatives under investigation and the standard, respectively. $\text{ I}_{\text{abs}}$ and $\text{ I}_{\text{abs}}^{\text{Std}}$ are the rates of light absorption by the Ps derivative and standard, respectively. *I_abs_* is determined by Eq. 3.

$I_{abs}= \frac{\alpha.A.I}{N_{A}}$ (3)

Where α is the fraction of light absorbed, A is the cell area irradiated, N_A_ is Avogadro’s constant and I is the light intensity.

1. **Supplementary Figures**


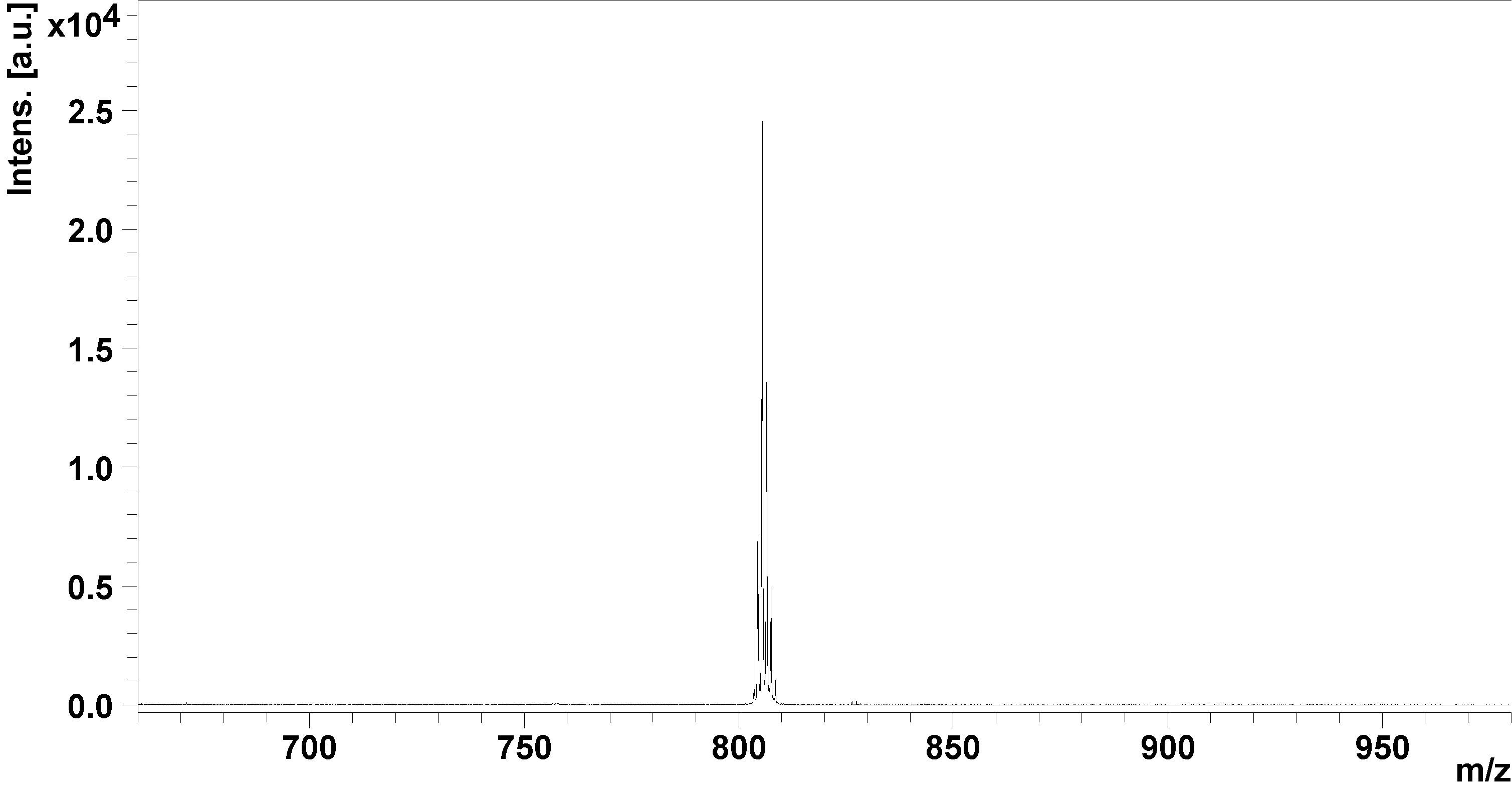


**[M]^+^ = 804.42**

**Supplementary Figure 1.** Mass spectrum of compound **1.**

**[M]^+^ = 866.61**

**Supplementary Figure 2.** Mass spectrum of compound **2.**

**[M]^+^ = 906.49**

**[M-Cl] ^+^= 871.48**

**Supplementary Figure 3.** Mass spectrum of compound **3.**

**[M+H]^+^ = 953.41**

**[M-Cl+H] ^+^= 918.38**

**Supplementary Figure 4.** Mass spectrum of compound **4.**

**Supplementary Figure 5.** ^1^H NMR compound **1** in DMSO-d6 (600 MHz).

**Supplementary Figure 6.** ^1^H NMR compound **3** in DMSO-d6 (600 MHz) as an example for the metalated Ps.

**Supplementary Figure 7.** Electronic absorption spectra of **2** and **2**-DNDs@Ag in DMSO.

**Supplementary Figure 8.** Electronic absorption spectra of **3** and **3**-DNDs@Ag in DMSO.

**Supplementary Figure 9.** The FT-IR spectra of (a)**1**, (b) **2**, (c) **3**, (d) **4**, (e) DNDs, (f) Ag NPs, (g) **1**-DNDs@Ag, (h) **2**-DNDs@Ag, (i) **3**-DNDs@Ag and (j) **4**-DNDs@Ag.

**Supplementary Figure 10.** The emission spectra of compounds in DMSO.

**(B)**

**(A)**

**(C)**

**Supplementary Figure 11**. Photobleaching of DMA in DMSO in the presence of (**A**) **1**-DNDS@Ag and (**B**) **2**-DNDs@Ag and (**C**) **4**-DNDs@Ag as examples.

1. **References**

Kee, H.L., Bhaumik, J., Diers, J.R., Mroz, P., Hamblin, M.R., Bocian, D.F., Lindsey, J.S., and Holten, D. (2008). Photophysical characterization of imidazolium-substituted Pd(II), in(III), and Zn(II) porphyrins as photosensitizers for photodynamic therapy. *J. Photochem. Photobiol. A: Chem*. 200, 346-355. doi.org/10.1016/j.jphotochem.2008.08.006.

Nyokong, T., Antunes, E., Kadish, K., Smith, K., and Guilard, R. (2010). (Eds.) Handb. *Porphyr. Sci*. World Scientific Publishing Co. Pte. Ltd. 247-349.

Ogunsipe, A., Chen, J.Y., and Nyokong, T. (2004). Photophysical and photochemical studies of zinc (II) phthalocyanine derivatives-effects of substituents and solvents. *New. J. Chem*. 7, 822-827. doi:10.1039/B315319C.
